# Interlayer excitons in a bulk van der Waals semiconductor

**DOI:** 10.1038/s41467-017-00691-5

**Published:** 2017-09-21

**Authors:** Ashish Arora, Matthias Drüppel, Robert Schmidt, Thorsten Deilmann, Robert Schneider, Maciej R. Molas, Philipp Marauhn, Steffen Michaelis de Vasconcellos, Marek Potemski, Michael Rohlfing, Rudolf Bratschitsch

**Affiliations:** 1 Institute of Physics and Center for Nanotechnology, University of Münster, Wilhelm-Klemm-Strasse 10, 48149 Münster, Germany; 2 Institute of Solid State Theory, University of Münster, Wilhelm-Klemm-Strasse 10, 48149 Münster, Germany; 3 Center for Atomic-Scale Materials Design (CAMD), Department of Physics, Technical University of Denmark, DK-2800 Kongens Lyngby, Denmark; 4 Laboratoire National des Champs Magnétiques Intenses, CNRS-UGA-UPS-INSA-EMFL, 25 rue des Martyrs, 38042 Grenoble, France

## Abstract

Bound electron–hole pairs called excitons govern the electronic and optical response of many organic and inorganic semiconductors. Excitons with spatially displaced wave functions of electrons and holes (interlayer excitons) are important for Bose–Einstein condensation, superfluidity, dissipationless current flow, and the light-induced exciton spin Hall effect. Here we report on the discovery of interlayer excitons in a bulk van der Waals semiconductor. They form due to strong localization and spin-valley coupling of charge carriers. By combining high-field magneto-reflectance experiments and ab initio calculations for 2H-MoTe_2_, we explain their salient features: the positive sign of the *g*-factor and the large diamagnetic shift. Our investigations solve the long-standing puzzle of positive *g*-factors in transition metal dichalcogenides, and pave the way for studying collective phenomena in these materials at elevated temperatures.

## Abstract (web summary)

Excitons, quasi-particles of bound electron-hole pairs, are at the core of the optoelectronic properties of layered transition metal dichalcogenides. Here, the authors unveil the presence of interlayer excitons in bulk van der Waals semiconductors, arising from strong localization and spin-valley coupling of charge carriers.

## Introduction

Monolayers of transition metal dichalcogenides (TMDCs) such as MoS_2_ are direct band gap semiconductors compared with their indirect gap multilayer counterparts. The hexagonal crystal structure gives rise to six energetically equivalent extrema in momentum space, which are alternatingly denoted as K^+^ and K^−^ valleys. Their electronic and optical properties are governed by strongly bound excitons, which exist at room temperature^1^. In monolayers, excitons are naturally confined to two dimensions (2D), i.e. a single layer. In a bilayer or a bulk system, a strong spin-layer locking exists^2–5^. Owing to this effect together with a weak van der Waals interlayer interaction, strongly confined electrons and holes might exist within different layers of a multilayer TMDC (interlayer excitons^6–9^), even without the requirement of a spacer layer^10, 11^. Interlayer excitons are potential candidates for unraveling a wealth of interesting physical phenomena, such as Bose-Einstein condensation, high temperature superfluidity, dissipationless current flow, and the light-induced exciton spin Hall effect^10–15^. Interlayer excitons have been successfully created in coupled GaAs quantum wells at cryogenic temperatures^16^. In contrast, TMDCs hold the promise for observing aforementioned phenomena at much higher temperatures^10, 11^. In TMDCs, interlayer excitons have been created in artificial heterostructures by stacking monolayers of two different TMDC materials on top of each other^6–9^. However, the present fabrication procedures are non-trivial. The rotation angle between the individual layers has to be precisely set^8^. Furthermore, coupling of the layers critically depends on the quality of the formed interfaces, which is difficult to control^17^.

Here, we show that interlayer excitons exist in an inversion-symmetric bulk TMDC semiconductor, 2H-MoTe_2_, which are accompanied by an inherent spin-layer locking. MoTe_2_ is a promising TMDC for novel electronic devices, because it has a band gap similar to silicon^18–20^ and extends the spectral range of TMDCs to the infrared region. Only recently, excitons in monolayer MoTe_2_ have been investigated using magneto-optical spectroscopy^21^.

## Results

### Optical spectroscopy of excitonic resonances

**Fig. 1:**
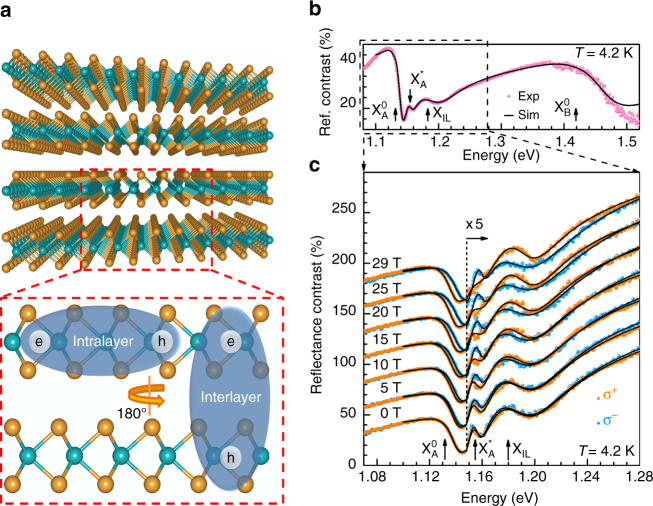
Crystal structure and magneto-reflectance spectra of bulk MoTe_2_. **a** Schematic drawing of the layer configuration in bulk 2H-MoTe_2_, highlighting intralayer and interlayer excitons. **b** Microreflectance contrast (μRC) spectrum of a 40 nm-thick 2H-MoTe_2_ crystal on SiO_2_(80 nm)/Si substrate in the absence of a magnetic field (*B* = 0) (*spheres*) together with the modeled spectrum (*solid line*). **c** Helicity-resolved μRC spectra at different magnetic fields *B* = 0–29 T (*spheres*) with the modeled spectra (*solid curves*). The spectra are vertically shifted with respect to 0 T spectrum for clarity

The micro-reflectance contrast (μRC) spectrum of a mechanically exfoliated 40 nm-thick bulk-like 2H-MoTe_2_ crystal on a SiO_2_(80 nm)/Si substrate is presented in Fig. 1b (see Methods section for details). Four prominent resonances are identified: ${\rm X}_{\rm{A}}^0$, ${\rm X}_{\rm{A}}^{\rm{*}}$, *X*_IL_, and ${\rm X}_{\rm{B}}^0$ with corresponding transition energies $E_{\rm{A}}^0 = 1.131 \pm 0.001$ eV, $E_{\rm{A}}^* = 1.155 \pm 0.002$ eV, ${E_{{\rm{IL}}}} = 1.183 \pm 0.003$ eV, and $E_{\rm{B}}^0 = 1.42 \pm 0.01$ eV, respectively. ${\rm X}_{\rm{A}}^0$ and ${\rm X}_{\rm{B}}^0$ correspond to the well-known A and B intralayer excitons with electron and hole confined to the same layer of bulk MoTe_2_ (Fig. 1a)^18^. As explained in the next sections, we assign ${\rm X}_{\rm{A}}^{\rm{*}}$ and *X*_IL_ to the ‘2*s*-like’ excited state of the intralayer A exciton and the ground state interlayer exciton, respectively.

**Fig. 2:**
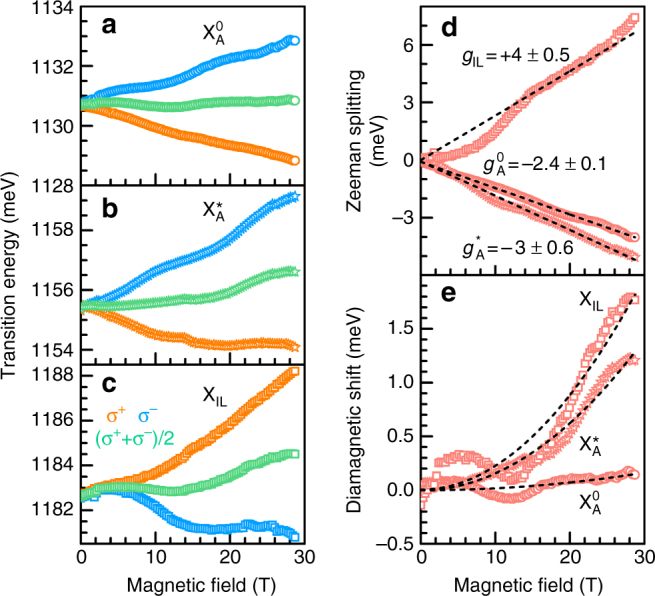
Excitonic Zeeman splittings and diamagnetic shifts. **a**–**c** Energies of the circularly polarized σ^+^ (*blue*) and σ^−^ (*orange*) optical transitions of the three exciton resonances ${\rm X}_{\rm{A}}^0$, ${\rm X}_{\rm{A}}^*$, and *X*_IL_ derived from the μRC spectra. Energies obtained by calculating the arithmetic mean of the *blue* and *orange* data are shown in *green*. **d** Zeeman splittings for the exciton resonances (*red points*). Note the reversal of the sign of the effective *g*-factor in **d** for the interlayer exciton *X*_IL_. **e** Diamagnetic shifts with fits to the data (*dashed lines*)

Figure 1c depicts helicity-resolved magneto-μRC spectra of MoTe_2_ under magnetic fields of up to *B* = 29 T. The σ^+^ and σ^−^ components of all excitonic resonances exhibit a Zeeman splitting Δ*E*, which increases linearly with rising magnetic field (Figs. 2a–d). ${{\Delta}}E = {E_{{\sigma ^ + }}}\!-\!{E_{{\sigma ^ - }}} = {g_{\rm{X}}}{\mu _{\rm{B}}}B$, where ${E_{{\sigma ^ + }}}$ and ${E_{{\sigma ^ - }}}$ are the excitonic transition energies for the two circular polarizations, *g*_X_ is the excitonic g-factor, and *μ*_B_ is the Bohr’s magneton. The excitonic resonances ${\rm X}_{\rm{A}}^0$ and ${\rm X}_{\rm{A}}^{\rm{*}}$ have negative *g*-factors of similar magnitude: $g_{\rm{A}}^0 = - 2.4 \pm 0.1$ and $g_{\rm{A}}^{2{{s}}} = - 3 \pm 0.6$, respectively. The similarity of these values suggests a common origin. Strikingly, the effective *g*-factor of *X*_IL_ is of opposite sign, with *g*_IL_ = +4 ± 0.5, indicating a different physical mechanism. Each excitonic resonance exhibits a diamagnetic shift depending on *B* ^2^: ${{\Delta}}E_{{\rm{dia}}}^{\rm{X}} = \left( {{e^2}{{\left\langle {{r_{\rm{X}}}} \right\rangle }^2}{B^2}} \right)/8\mu _{\rm{X}}^{\rm{*}}$, with *e* the electronic charge, $\left\langle {{r_{\rm{X}}}} \right\rangle$ the excitonic root-mean-square (RMS) radius, and $\mu _{\rm{X}}^*$ the reduced mass^22^. ${{\Delta}}E_{{\rm{dia}}}^{\rm{X}}$ is the deviation of the exciton’s mean transition energy $\left( {{E_{{\sigma ^ + }}} + {E_{{\sigma ^ - }}}} \right)/2$ for $B \,\ne\, 0$ from the zero-field value. Figure 2e reveals that ${\rm X}_{\rm{A}}^0$ shows a significantly smaller diamagnetic shift (${{\Delta}}E_{{\rm{dia}}}^0 = 0.2 \pm 0.1{{\rm{T}}^{ - 1}}$) compared with those for ${\rm X}_{\rm{A}}^{\rm{*}}$ (${{\Delta}}E_{{\rm{dia}}}^* = 1.6 \pm 0.1$ μeVT^−1^) and ${X_{{\rm{IL}}}}\left( {{{\Delta}}E_{{\rm{dia}}}^{{\rm{IL}}} = 2.3 \pm 0.1\,{\rm{\mu e}}V\,{{\rm{T}}^{ - 1}}} \right)$. This observation points towards relatively large RMS radii and smaller excitonic binding energies for ${\rm X}_{\rm{A}}^{\rm{*}}$ and *X*_IL_ compared with ${\rm X}_{\rm{A}}^0$.

### Identification of interlayer excitons

**Fig. 3:**
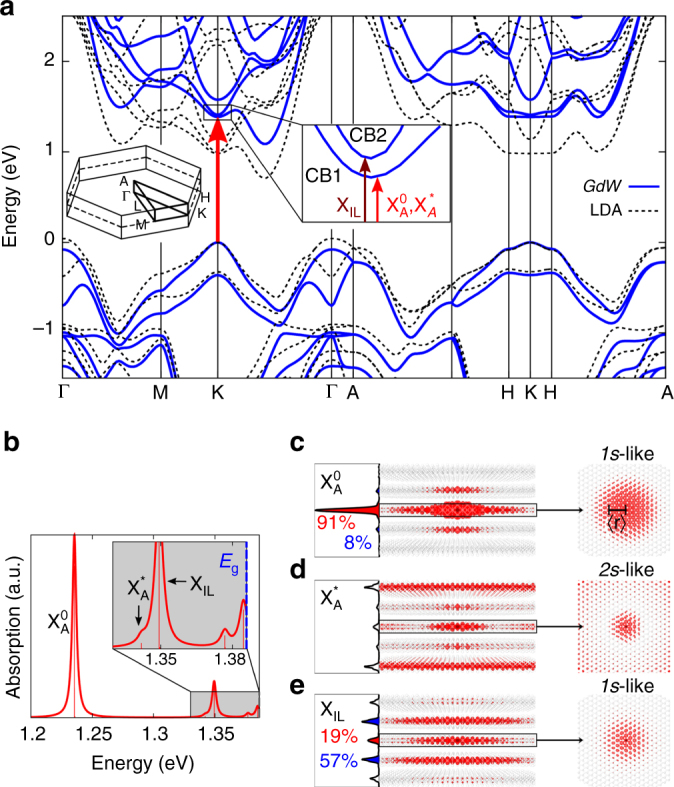
Calculated exciton absorption spectra and spatial distributions. **a** LDA (local density approximation) (*dashed line*) and quasiparticle band structure in the *GdW* approximation (*solid blue line*) of bulk MoTe_2_. *Vertical arrows* indicate the largest contributions from the bands to the ${\rm X}_{\rm{A}}^0$, ${\rm X}_{\rm{A}}^{\rm{*}}$, and *X*_IL_ excitations at the K point. The *left inset* shows the three-dimensional Brillouin zone. **b** Calculated exciton absorption spectrum using a linewidth broadening of 2 meV with a zoomed-in view on the ${\rm X}_{\rm{A}}^{\rm{*}}$ and *X*_IL_ resonances. The *dashed blue line* at 1.385 eV indicates the direct band gap at K. **c** *Side view* (*left*) and *top view* (*right*) of the spatial distribution of the three excitons in the bulk crystal. The integrated probabilities of the excitonic spread are plotted for each layer *left* of the *side view*. All calculations are performed in absence of a magnetic field

To confirm the identification of the observed resonances, we calculate the quasiparticle band structure and the optical absorption spectrum for bulk 2H-MoTe_2_ using the *GW*–Bethe–Salpether equation (BSE) method within the LDA + *GdW* approximation^23^ (see Methods section for details). The quasiparticle band structure is represented by solid lines in Fig. 3a. Our calculated valence bands (VBs) are in good agreement with the results of angle-resolved photoemission spectroscopy measurements performed for 2H-MoTe_2_.^24^ The calculated direct and indirect band gaps are 1.39 and 1.09 eV. A flat dispersion along the *k*_*z*_ (or K–H) direction around the K point implies a large out-of-plane effective mass and strong in-plane carrier confinement, which demonstrates the 2D character of the charge carriers even for bulk material. In the calculated absorption spectrum (Fig. 3b), we find that the first three low-energy optical transitions originate from the *K* point of the Brillouin zone. The first resonance at 1.24 eV corresponds to the intralayer exciton ${\rm X}_{\rm{A}}^0$ (binding energy $E_{\rm{b}}^0 = 150$ meV), which is in good agreement with the experimental transition energy of 1.131 eV. Figure 3c shows the spatial distribution for ${\rm X}_{\rm{A}}^0$, obtained by fixing the positive hole in the vicinity of a Mo atom and calculating the probability distribution of the electron around it. Strikingly, this exciton with a calculated RMS radius $\left\langle {{r_0}} \right\rangle$  = 1.2 nm is strongly confined (>90%) within a single layer, demonstrating its 2D excitonic character. From the experimentally observed value for the diamagnetic shift (see methods), we determine $\left\langle {{r_0}} \right\rangle$ = 1.7 ± 0.4 nm, which is in good agreement with the calculated value. The next two resonances at higher energies, 1.3 and 1.35 eV (Fig. 3b), are identified as the ${\rm X}_{\rm{A}}^{\rm{*}}$ and X_IL_ features observed in Fig. 1b. The calculated relative oscillator strengths of these three transitions in increasing order of energy are 1, 0.07, and 0.51, respectively. Experimentally, we obtain 1, 0.01, and 0.19, which reasonably agrees with the calculated trend. The spatial distribution of the resonance at 1.34 eV has a ‘2*s*-like’ excitonic character (*top view* in Fig. 3d), verifying its assignment as ${\rm X}_{\rm{A}}^{\rm{*}}$. The RMS radius obtained from the experimentally derived diamagnetic shift is $\left\langle {{r_*}} \right\rangle$ = 4.8 ± 0.4 nm. ${\rm X}_{\rm{A}}^{\rm{*}}$ is also spatially extended to the third layer (Fig. 3d). This is in qualitative agreement with the radial probability distribution of the 2 s orbital of the H atom, where one expects a large extent of the wave function (see Supplementary Fig. 3). As we find that both ${\rm X}_{\rm{A}}^0$ and ${\rm X}_{\rm{A}}^{\rm{*}}$ are related to the same interband transitions, we expect their *g*-factors to be similar, which is indeed observed in the experiment. It must be noted that in the *GW*-BSE approach it is difficult to converge the details of the absorption spectrum near the direct band gap at 1.386 eV. Both the dense Rydberg series just below the band gap, as well as the onset (at the gap) of a Coulomb-enhanced continuous spectrum, require many more *k*-points than used in our study and could better be described in an appropriate Wannier exciton model with appropriate parameters. Therefore, we concentrate on the well-converged discrete exciton states below 1.37 eV.

Strikingly, for the 1.35 eV resonance (*X*_IL_), with the hole fixed in one layer, the probability of finding an electron within the same layer is much smaller ( ~ 19%) than in the neighboring layers ( ~ 57%) (Fig. 3e), demonstrating its interlayer nature. It is weakly bound (calculated $E_{\rm{b}}^{{\rm{IL}}} = 60$ meV) compared with the intralayer exciton. In addition, the RMS radii obtained for *X*_IL_ from our ab initio calculations and the one using the diamagnetic shift are $\left\langle {{r_{{\rm{IL}}}}} \right\rangle$ ~ 2.0 nm and $\left\langle {{r_{{\rm{IL}}}}} \right\rangle = 5.0 \pm 0.4\,{\rm{nm}}$, respectively. Although these values differ significantly, possibly due to errors in the computed exciton reduced mass and in the exciton wave function with respect to the used *k*-mesh (see Methods), they overall point towards a larger spread of this state in real-space. This is consistent with the weakening of the Coulomb binding for a large spatial separation of electron and hole for X_IL_^10^. Nevertheless, the binding energy of the bulk TMDC interlayer exciton $E_{\rm{b}}^{{\rm{IL}}}$ is almost an order of magnitude larger compared with those in coupled quantum wells^25^, which renders the existence of bulk TMDC interlayer excitons possible at elevated temperatures (Supplementary Fig. 6) or even at room temperature.

## Discussion

**Fig. 4:**
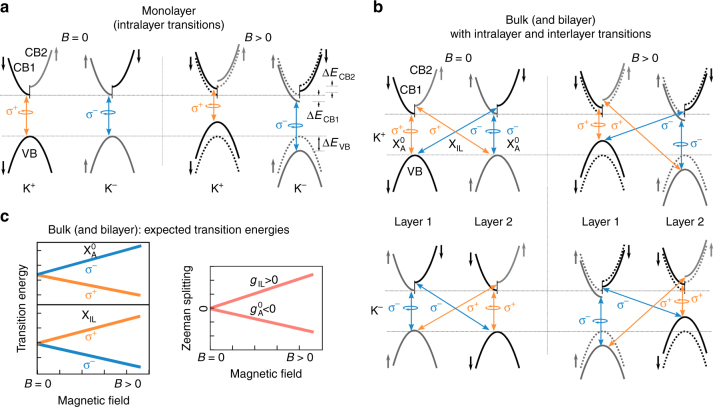
Transition selection rules for intra- and interlayer excitons. Helicity-resolved optical selection rules for the transitions at *B* = 0 (*left*) and *B* > 0 (*right*) between the valence band VB and the spin–orbit–split conduction bands CB1 and CB2 (with carrier spins marked) in the case of **a** monolayer and **b** bulk (and bilayer) 2H-MoTe_2_. The *dashed lines* in the *right panels* indicate the band positions for *B* = 0. *Vertical* and *diagonal arrows* indicate intralayer (${\rm X}_{\rm{A}}^0$) and interlayer transitions (*X*_IL_). **c** Theoretically expected trends of the excitonic transition energies and the Zeeman splittings for the intralayer (${\rm X}_{\rm{A}}^0$) and interlayer (*X*_IL_) excitons

To understand the magneto-optical transitions of the intralayer and interlayer excitons, we derive the interband transition selection rules for circularly polarized light by calculating the circular optical dipole matrix elements between the bands^2^ (see Supplementary Fig. 4). The well-established case of monolayer 2H-MoTe_2_^21^ is presented in the *left panel* of Fig. 4a. The spin of the electron or hole is locked to the valley and is conserved during an optical transition^26^. For *B* = 0, the σ^+^ and σ^−^ transitions occur in the K^+^ and K^−^ valleys, respectively, which are degenerate in energy. For *B* > 0 (*right panel* of Fig. 4a) the valley degeneracy is lifted^21^. The energy of the σ^+^ (σ^−^) transition decreases (increases), giving rise to a negative excitonic *g*-factor.

For a bilayer or bulk TMDC, every band is doubly degenerate with opposite spin configurations due to inversion and time-reversal symmetry. For the 2H polytype, adjacent layers are rotated by 180° (Fig. 1a), which results in an inversion of the spin orientation when going from one layer to the next (“spin-layer locking”)^2–5^ in all bands. For instance, for the *K* ^+^ valley, the VB and the CB1 for layer 1 (layer 2) have spin down (up) character (*top row* of Fig. 4b). The 2D confinement of excitons within the individual neighboring layers of bulk material results in spatially direct (intralayer) transitions (*vertical arrows* in Fig. 4b). These transitions are similar to the monolayer case, except that for a given valley, the σ^+^ and σ^−^ transitions take place in adjacent layers (see Supplementary Fig. 5a, e for calculated transition selection rules). The *g*-factor of the intralayer transitions in bulk has a negative sign and similar magnitude (see Fig. 4c for ${\rm X}_{\rm{A}}^0$), reaffirming their monolayer-like 2D character. In contrast, the high-energy spin-orbit-split conduction band CB2 participates in the formation of interlayer transitions (*diagonal arrows* in Fig. 4b). The carrier spins for the σ^+^ and σ^−^ interlayer transitions are reversed when compared with the corresponding intralayer transitions. The calculated transition selection rules for this case are presented in Supplementary Fig. 5d, h. For *B* > 0, the interlayer σ^+^ transitions increase in energy compared with the *B* = 0 case, whereas the σ^−^ transitions decrease in energy (Fig. 4b, *right panel*) giving rise to a positive *g*-factor (see Fig. 4c for X_IL_). The contribution from the orbital magnetic moment to interlayer excitons is expected to be similar to intralayer excitons, but with opposite sign, i.e.,  + 4, which is consistent with our measured value of  + 4 ± 0.5.

In summary, we have performed magneto-reflectance contrast spectroscopy under high magnetic fields and *GW*-BSE ab initio calculations to unveil interlayer excitons in the bulk van der Waals semiconductor 2H-MoTe_2_. The interlayer excitons form due to strong localization and spin-valley coupling of the charge carriers and are characterized by *g*-factors with opposite sign and large diamagnetic shift compared with intralayer excitons. Interestingly, unexplained positive *g*-factors have been reported also for bulk MoSe_2_ and MoS_2_ using magnetic circular dichroism spectroscopy^27^, which suggests that interlayer excitons might also exist in these bulk TMDCs. Indeed, our *GW*-BSE calculations indicate that bulklike MoS_2_ and MoSe_2_ also exhibit interlayer excitons similar to MoTe_2_ (Supplementary Fig. 7).

## Methods

### Experiment

For micro-magneto-reflectance (μRC) measurements, an optical fiber-based low-temperature probe is placed inside a 50 nm-diameter bore of a resistive magnet, which generates magnetic fields up to 29 T at the center of the magnet. A 50 μm-diameter optical fiber carries unpolarized light to the sample, which is placed on *x*–*y*–*z* piezo-nanopositioners. The light used for excitation is focused on the sample using an aspheric lens of focal length 3.1 mm and a numerical aperture of 0.68. Light reflected from the sample is circularly polarized in situ using a combination of a quarter wave plate and a polarizer. The polarized light is collected using a 200 μm diameter optical fiber and detected with a monochromator and an InGaAs array. The measurements are performed using a fixed circular polarization configuration of the quarter wave plate-polarizer assembly, whereas the magnetic field is reversed to acquire spectral information corresponding to the other direction of circular polarization due to time-reversal symmetry^28^. The reflectance contrast spectrum *C*(*λ*) is defined as $C\left( \lambda \right) = \left[ {R\left( \lambda \right) - {R_0}\left( \lambda \right)} \right]/\left[ {R\left( \lambda \right) + {R_0}\left( \lambda \right)} \right]$, where *R*(*λ*) and *R*_0_(*λ*) denote the reflectance of the MoTe_2_ crystal on the substrate and the bare substrate, respectively, for a given wavelength *λ*. μRC spectral lineshapes are modeled using a transfer matrix approach for obtaining the transition energies (solid lines in Fig. 1c, d). The excitonic contribution to the dielectric response function is calculated by a Lorentz oscillator model^29^

1 $${\it{\epsilon }}\left( E \right) = {\left( {{n_{\rm{b}}} + {\rm{i}}{k_{\rm{b}}}} \right)^2} + \frac{A}{{E_0^2 \!-\! {E^2} \!-\! {\rm{i}}\gamma E}}$$

where *n*_b_+i*k*_b_ is the background complex refractive index of MoTe_2_ in the absence of excitonic effects, and is assumed to be equal to that of bulk material^30^. *E*_0_ is the transition energy, *A* is the oscillator strength parameter, and *γ* is the full width at half maximum line width. The oscillatory features in the data of Fig. 2 are due to Faraday rotation effects of a small linearly polarized component (<10%) in detection^21^.

### Calculations

In the density functional theory calculations, we employ a basis of three shells of localized Gaussian orbitals with s, p, d and s* symmetry and decay constants (in $a_B^{ - 2}$) of [0.14, 0.4, 1.1] for Te and [0.16, 0.51, 1.42] for Mo along with a *k*-mesh of 12 × 12 × 3 points. The *GW* calculations are conducted in the *GdW* approximation to the self-energy, where an atom-resolved dielectric model function based on the random-phase approximation is used for representing the dielectric screening properties^23^. We use the experimentally obtained lattice structure of 2H-MoTe_2_ from ref. ^24^. The energy cutoff of the auxiliary plane wave basis is set to 2.5 Ry (see Supplementary Fig. 1 for energy gap convergence as a function of cutoff energy and the *k*-mesh). Figure 3b depicts the calculated absorption spectrum resulting from the solution of the BSE equation using a 30 × 30 × 4 *k*-grid in the first Brillouin zone and four valence and eight conduction bands (see Supplementary Fig. 2 for convergence of transition energies as a function of *k*-mesh). The used meshes are identical for the electron-hole interaction and the quasiparticle band structure so no interpolation is needed. Spin–orbit interaction is fully accounted for during all computational steps.

The energetic separation of the two resonances ${\rm X}_{\rm{A}}^{\rm{*}}$ and *X*_IL_ from ${\rm X}_{\rm{A}}^0$ are 24 and 52 meV in the experiment (Fig. 1b), whereas we find 100 and 110 meV in the calculation. The two main sources of discrepancy in the theoretical description (apart from the methodological error) are the limits of convergence of our calculation for such small energies, and experimental uncertainties in the determination of the lattice structure we used as input for our calculation, which is discussed as follows: (1) the numerical convergence limit, which depends mostly on the used *k*-mesh density and the number of bands included in the BSE Hamiltonian, as represented by the Supplementary Figs. 1 and 2. The *k*-mesh convergence for a 2*s*-like state ${\rm X}_{\rm{A}}^{\rm{*}}$ is especially demanding due to a large spatial extent and internal structure of the wave function that needs to be well captured^31^. (2) We use the experimental lattice structure^24^ for the covalent bonds as well as the interlayer separation, which is difficult to calculate within density functional theory (DFT) due to the van der Waals interaction. As an alternative, we have relaxed the covalent bonds within each layer and only use the experimental lattice constant for the layer separation. We found that the energetic separation between $X_{\rm{A}}^0$ and $X_{\rm{A}}^{\rm{*}}$ critically depends on the details of the lattice structure. For instance, the relaxed in-plane lattice constant is merely 0.1% larger than the experimental one, but the energetic difference between ${\rm X}_{\rm{A}}^0$ and ${\rm X}_{\rm{A}}^{\rm{*}}$ reduces from 100 to 80 meV.

The RMS radius of the exciton is calculated using $\sqrt {\left\langle {r_{\rm{X}}^2} \right\rangle } = {\left[ {\frac{{\left\langle {{\Phi ^{\rm{X}}}\left| {{{\left( {{{\bf{r}}_{\bf{e}}} - {{\bf{r}}_{\bf{h}}}} \right)}^2}} \right|{\Phi ^{\rm{X}}}} \right\rangle }}{{\left\langle {{\Phi ^{\rm{X}}}|{\Phi ^{\rm{X}}}} \right\rangle }}} \right]^{\frac{1}{2}}}$, where ${\Phi ^{\rm{X}}}$ denotes the three-dimensional electron distribution in real space (i.e., the exciton wave function with a fixed hole), and |**r**_**e**_−**r**_**h**_| is the distance between the electron and the hole. When evaluating the RMS radius on a grid, special care has to be taken since the electron distribution repeats itself, e.g., after 30 × 30 × 4 lattice vectors for a *k*-mesh of 30 × 30 × 4. To avoid double counting, a cutoff radius for the evaluation in real space is introduced. The charge-carrier effective masses used for determining the Bohr radius from experimentally obtained diamagnetic shifts are calculated using the LDA + *GdW* band structure^23^. This is done by fitting parabolas in an area of $0.03\frac{\pi }{a}$ at the band extrema and calculating the band curvature. The values of the effective masses are $m_{{\rm{CB}}1}^* = 0.83;m_{{\rm{CB}}2}^* = 0.44; m_{{\rm{VB}}}^* = 0.56$ (in units of electron mass), where CB1 and CB2 are the lower and higher energy spin–orbit–split conduction bands in Fig. 3, and VB is the topmost valence band at the K point. Although CB1 participates in the creation of intralayer excitons ${\rm X}_{\rm{A}}^0$ and ${\rm X}_{\rm{A}}^{\rm{*}}$, CB2 is involved in the formation of interlayer excitons X_IL_. It must be noted that the used effective masses are an average over the effective mass in KГ and KM direction. The *GdW* approximation in the quasiparticle band structure, the fitting procedure and the mass averaging increase the uncertainty of the calculated effective masses and hence of the RMS radii derived from experiment.

In Fig. 4 we schematically present the circular-polarization-resolved optically allowed transitions after calculating the optical dipole matrix elements between the conduction and VBs. The calculations are performed for monolayer and bulk MoTe_2_. The spin orientations of the bands are determined from the expectation values of the spin polarizations of the DFT wave functions. The solution of the BSE Hamilton operator provides the absorption spectrum with exciton energies, along with the wave function of every exciton. The real space wave functions for ${\rm X}_{\rm{A}}^0$, ${\rm X}_{\rm{A}}^{\rm{*}}$, and *X*_IL_ are depicted in Figs. 3c–e. The wave functions in *k*-space are used to determine which bands and *k*-points contribute the most to the excitation at hand. For example the interlayer exciton X_IL_ involves a transition mainly at the K point between the (degenerate) topmost VB and the degenerate CB1 (see Fig. 3a).

The polarization of excitations between a valence and a conduction band can be obtained using the optical dipole matrix elements. We calculate the degree of circular polarization, *η*(***k***), which is defined by $\eta \left( {\boldsymbol{k}} \right) = \frac{{{{\left| {{\cal P}_{{\rm{cv}}}^ + } \right|}^2} - {{\left| {{\cal P}_{{\rm{cv}}}^ - } \right|}^2}}}{{{{\left| {{\cal P}_{{\rm{cv}}}^ + } \right|}^2} + {{\left| {{\cal P}_{{\rm{cv}}}^ - } \right|}^2}}}$, where ${\cal P}_{{\rm{cv}}}^ \pm = \frac{1}{{\sqrt 2 }}\left[ {{\cal P}_{{\rm{cv}}}^x \pm i{\cal P}_{{\rm{cv}}}^y} \right]$, with ${\cal P}_{cv}^{x/y}$ as the interband matrix elements of the dipole operator evaluated with the LDA–DFT wave functions. The results of the calculations are shown in Supplementary Fig. 4 for the monolayer, and Supplementary Fig. 5 for the bulk system.

### Data availability

The data that support the findings of this study are available from the corresponding author upon reasonable request.

## Electronic supplementary material

Supplementary Information
