# Publisher Correction: Interlayer excitons in a bulk van der Waals semiconductor

**DOI:** 10.1038/s41467-017-01621-1

**Published:** 2017-11-17

**Authors:** Ashish Arora, Matthias Drüppel, Robert Schmidt, Thorsten Deilmann, Robert Schneider, Maciej R. Molas, Philipp Marauhn, Steffen Michaelis de Vasconcellos, Marek Potemski, Michael Rohlfing, Rudolf Bratschitsch

**Affiliations:** 1 Institute of Physics and Center for Nanotechnology, University of Münster, Wilhelm-Klemm-Strasse 10, 48149 Münster, Germany; 2 Institute of Solid State Theory, University of Münster, Wilhelm-Klemm-Strasse 10, 48149 Münster, Germany; 3 Center for Atomic-Scale Materials Design (CAMD), Department of Physics, Technical University of Denmark, DK-2800 Kongens Lyngby, Denmark; 4 Laboratoire National des Champs Magnétiques Intenses, CNRS-UGA-UPS-INSA-EMFL, 25 rue des Martyrs, 38042 Grenoble, France

*Nature Communications* **8**:639 doi:10.1038/s41467-017-00691-5; Article published online: 21 September 2017

In the PDF version of this article, Equation 1 is missing an epsilon at the beginning. The correct version of Equation 1 is below. The HTML version of the paper was correct from the time of publication.

$$\epsilon \left( E \right) = \left( {n_{\mathrm{b}} + {\mathrm{i}}k_{\mathrm{b}}} \right)^2 + \frac{A}{{E_0^2 - E^2 - {\mathrm{i}}\gamma E}}.$$

