# Supplementary material for: Interlayer excitons in a bulk van der Waals semiconductor
Source: Nat Commun. 2017 Sep 21;8:639. doi: 10.1038/s41467-017-00691-5 (PMC5608874; doi:10.1038/s41467-017-00691-5)
Supplement: Supplementary file 1 — Supplementary Information [file 41467_2017_691_MOESM1_ESM.pdf]

Description of Supplementary Files

File Name: Supplementary Information
Description: Supplementary Figures

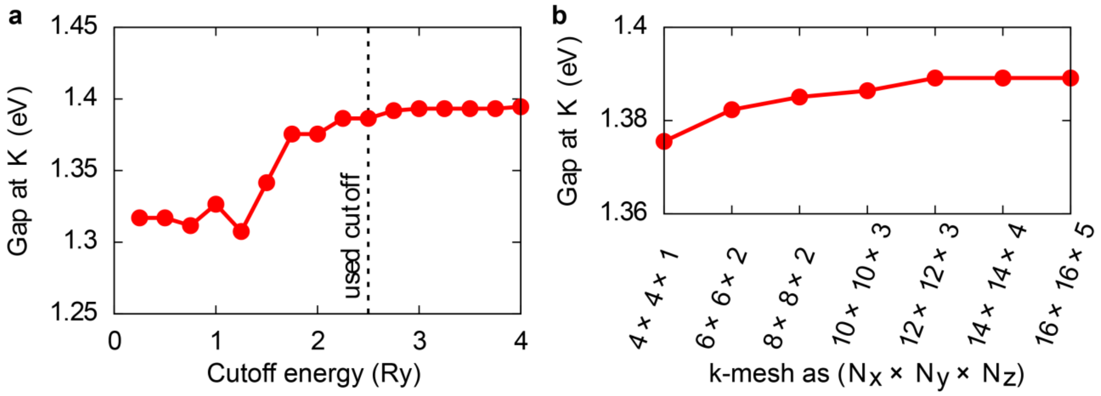

Supplementary Figure 1. Convergence of quasiparticle band gap. Convergence of the direct quasiparticle band gap at K as a function of the cutoff energy of the auxiliary plane wave basis (with a fixed 10×10×3 k-mesh) in (A), and as a function of the k-mesh in the first Brillouin zone (with a fixed cutoff energy of 2.5 Ry) in (B). Note the fast convergence of the LDA+*GdW* quasiparticle gap: all shown gap values are spread in energy by about 0.1 eV in (A) and about 0.02 eV in (B).

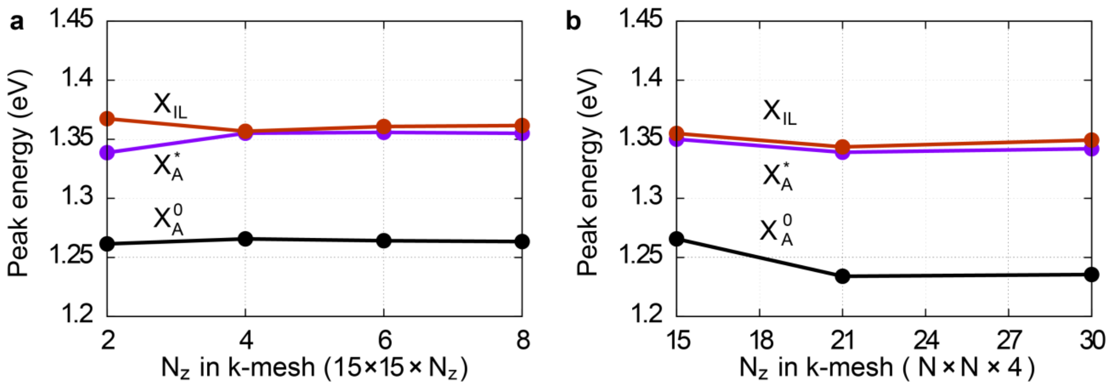

Supplementary Figure 2. Convergence of *GW*-BSE transition energies. Convergence of the three lowest optically active excitons (those discussed in detail in the main text) with respect to with respect to N_z using a fixed $15 \times 15 \times N_z$ mesh in (**a**), and with respect to N in $N \times N \times 4$ in (**b**). Neither changing the number of k-points in the *z* direction (**a**), nor in the *x* and *y* direction (**b**) leads to a reordering of the excitations. The lowest excitation X_A^0 shows almost no dependence on N_z.

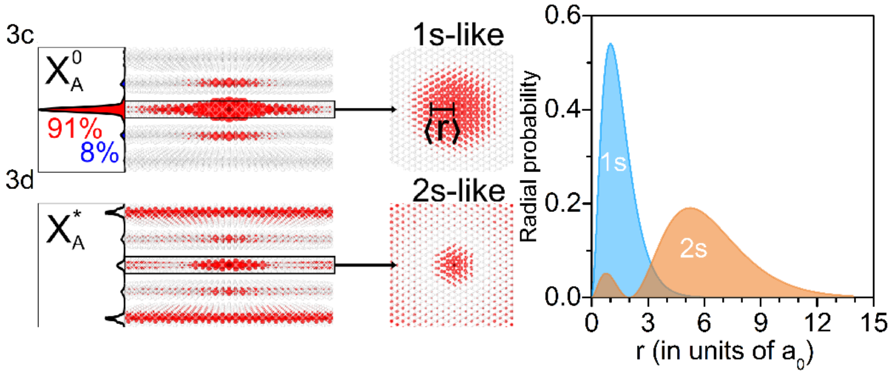

Supplementary Figure 3. Exciton versus H-atom spatial probability distribution. (Left) Reproduction of Fig. 3c and 3d from the main text, (right) Radial probability distribution of electron in 1s and 2s orbitals of a Hydrogen atom as a function of distance r, in units of the Bohr radius a_0. The 2s-like state X_A^* of $MoTe_2$ has significant spatial spread to the third layer from the center in 3d. This is qualitatively similar to the H-atom's 2s state probability distribution in the right panel, which is significant within the second maximum of the distribution away from the center $r = 0$.

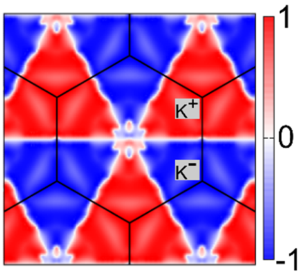

Supplementary Figure 4. Degree of polarization in monolayer $MoTe_2$. Calculated degree of circular polarization between the VB and the CB1 in monolayer $MoTe_2$. At the K^+ (K^-) point, excitons can be created using right (left) handed polarized light.

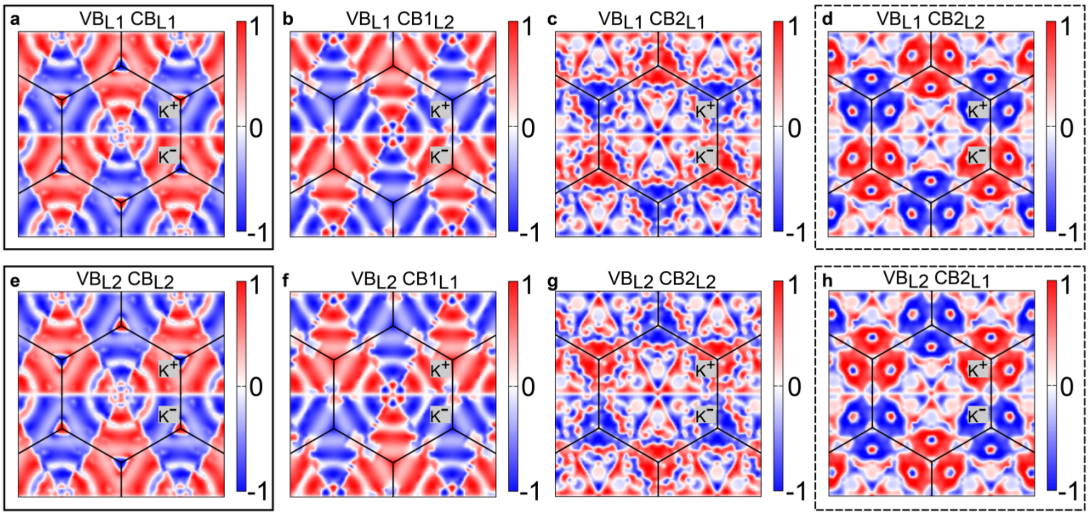

Supplementary Figure 5. Degree of polarization in bulk $MoTe_2$. Calculated degree of circular polarization $\eta(\mathbf{k})$ in bulk $MoTe_2$ between the top valence band VB and the lowest two conduction bands CB1 and CB2. All bands are two-fold degenerate, due to the two different layers in the unit cell denoted as L1 and L2. Only the $k_z = 0$ plane is shown, which is where the optical dipole matrix elements are by far the largest. As an example, the intralayer excitation between VB_{L1} (valence band in layer 1) and $CB1_{L1}$ (bottommost conduction band in layer 1) in (**a**) is right (left) circularly polarized at K^+ (K^-) point. On the other hand, the interlayer transition between VB_{L1} and $CB2_{L2}$ in (**d**) is left (right) circularly polarized at $K^+(K^-)$ points. It must be noted that the degree of polarization depicted here does not carry information about the magnitudes of the oscillator strengths of the transitions and thus does not indicate on its own whether a transition is optically bright or dark. The plots enclosed by the solid (intralayer exciton X_A^0) and dashed (interlayer exciton X_{IL}) boxes correspond to the optically bright transitions, whereas the other four plots represent optically dark excitons. It is worthwhile mentioning that among the optically bright transitions, the intralayer excitons possess larger oscillator strength than the interlayer excitons.

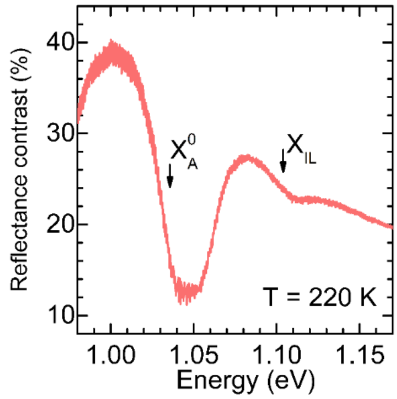

Supplementary Figure 6. Observation of X_{IL} at elevated temperatures. Reflectance contrast spectrum of a 25 nm thick bulk-like $MoTe_2$ crystal measured at a temperature T = 220 K in the absence of a magnetic field. The intralayer A exciton X_A^0 and the interlayer exciton X_{IL} can be identified.

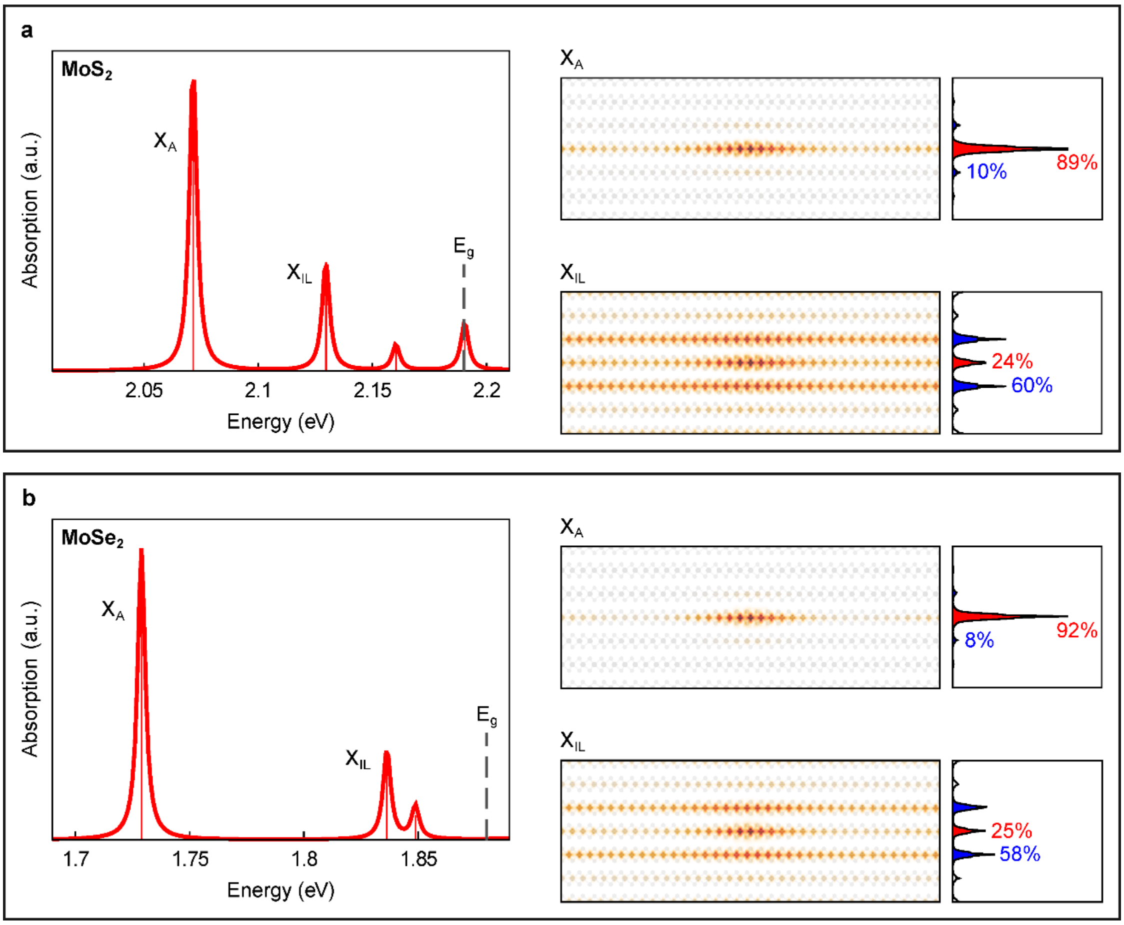

Supplementary Figure 7. Interlayer excitons in bulk MoS_2 and $MoSe_2$. Results of *GW*-BSE calculations on bulklike (**a**) MoS_2, and (**b**) $MoSe_2$. Left panel: Low energy absorption spectrum showing the X_A^0 exciton and the interlayer exciton X_IL below the direct gap E_g (at the K point). A side view of the excitons' spatial distributions is displayed on the right (in analogy to Fig. 3 of the main text). The hole is fixed in the center of the middle layer. On the far right panel, the probability to find the electron in the respective layers is integrated, which clearly indicates the interlayer character of X_IL. A k-mesh of $18 \times 18 \times 3$ is used for the calculations.
